# Estimating the impact of non-pharmaceutical interventions against COVID-19 on mumps incidence in Sichuan, China

**DOI:** 10.1186/s12879-021-06584-9

**Published:** 2021-08-30

**Authors:** Wenqiang Zhang, Rongsheng Luan

**Affiliations:** Department of Epidemiology and Health Statistics, West China School of Public Health and West China Fourth Hospital, Sichuan University, Chengdu, 610041 Sichuan China

**Keywords:** COVID-19, Mumps, Mathematical model, Non-pharmaceutical interventions

## Abstract

**Background:**

A series of social and public health measures have been implemented to contain coronavirus disease 2019 (COVID-19) in China. We examined the impact of non-pharmaceutical interventions against COVID-19 on mumps incidence as an agent to determine the potential reduction in other respiratory virus incidence.

**Methods:**

We modelled mumps incidence per month in Sichuan using a seasonal autoregressive integrated moving average (ARIMA) model, based on the reported number of mumps cases per month from 2017 to 2020.

**Results:**

The epidemic peak of mumps in 2020 is lower than in the preceding years. Whenever compared with the projected cases or the average from corresponding periods in the preceding years (2017–2019), the reported cases in 2020 markedly declined (*P* < 0.001). From January to December, the number of mumps cases was estimated to decrease by 36.3% (33.9–38.8%), 34.3% (31.1–37.8%), 68.9% (66.1–71.6%), 76.0% (73.9–77.9%), 67.0% (65.0–69.0%), 59.6% (57.6–61.6%), 61.1% (58.8–63.3%), 49.2% (46.4–52.1%), 24.4% (22.1–26.8%), 30.0% (27.5–32.6%), 42.1% (39.6–44.7%), 63.5% (61.2–65.8%), respectively. The total number of mumps cases in 2020 was estimated to decrease by 53.6% (52.9–54.3%).

**Conclusion:**

Our study shows that non-pharmaceutical interventions against COVID-19 have had an effective impact on mumps incidence in Sichuan, China.

**Supplementary Information:**

The online version contains supplementary material available at 10.1186/s12879-021-06584-9.

## Background

The first wave of coronavirus disease 2019 (COVID-19) in China has been addressed with the implementation of substantial public health measures [1–3]. These measures referring to social distancing, contact tracking, quarantine, isolation, and personal protection, are indispensable components of the public health response to COVID-19, which contribute to containing COVID-19 transmission [3–5]. Whether non-pharmaceutical interventions against COVID-19 would reduce other respiratory virus transmission has been noticed.

Previous studies have shown that non-pharmaceutical interventions against COVID-19 were associated with the reduction of influenza cases [4, 6–8]. Influenza and mumps have similarities in the modes of transmission through contact and droplet spread [9]. Mumps has been the most common infection in children and adolescents in China [10]. However, whether non-pharmaceutical interventions against COVID-19 would reduce the mumps incidence is lacking. Based on the reported mumps cases in Sichuan, we developed a seasonal autoregressive integrated moving average (ARIMA) model to estimate the impact of non-pharmaceutical interventions against COVID-19 on mumps incidence as an agent to determine the potential reduction in other respiratory virus incidence.

## Methods

### Data source

We obtained data from the official website of Health Commission of Sichuan Province (http://wsjkw.sc.gov.cn/). The number of mumps cases per month was reported from January 2017 to July 2020.

### Non-pharmaceutical interventions against COVID-19

The first confirmed COVID-19 case from Wuhan in Sichuan was reported on January 21, 2020, and emergency response to major public health events in Sichuan upgraded to level 1 (the highest level) on January 24, 2020. A series of non-pharmaceutical interventions against COVID-19 were implemented, including social distancing, contact tracking, personal protection, travel restriction, etc. First, school holidays were extended, and schools were not allowed to open without permission of the local government, especially the kindergarten and primary school. Second, mass gatherings and major public events were canceled, and people were encouraged to stay at home as much as possible. Third, personal protective behaviors became normalized, such as hand washing and face masks. Additionally, the local government organized environmental disinfection in public places. Continuous rigorous social and public health measures could effectively decrease the spread of mumps.

### Model construction

We modelled mumps incidence per month in Sichuan using the seasonal autoregressive integrated moving average (ARIMA) model, ARIMA (*p*, *d*, *q*) × (*P*, *D*, *Q*) _*S*_ given by:

$$\begin{gathered} \nabla^{d} \nabla_{S}^{D} x_{t} = \frac{{\Theta (B)\Theta_{S} (B)}}{{\Phi (B)\Phi_{S} (B)}}\varepsilon_{t} \hfill \\ \Theta (B) = 1 - \theta_{1} B - \cdots - \theta_{q} B^{q} \hfill \\ \Phi (B) = 1 - \phi_{1} B - \cdots - \phi_{p} B^{p} \hfill \\ \Theta_{S} (B) = 1 - \theta_{1} B^{S} - \cdots - \theta_{Q} B^{QS} \hfill \\ \Phi_{S} (B) = 1 - \phi_{1} B^{S} - \cdots - \phi_{P} B^{PS} \hfill \\ \end{gathered}$$

where *p* is the AR order, *d* is the degree of differencing, *q* is the MA order; *P* is the seasonal AR order, *D* is the degree of seasonal differencing, *Q* is the seasonal MA order, and *S* is the length of the seasonal period; *B* is the backshift operator. We used a seasonal-trend decomposition procedure based on Loess (STL) to decomposing a time series into trend, seasonal, and remainder components (Additional file 1: Figure S1) [11].

**Fig. 1:**
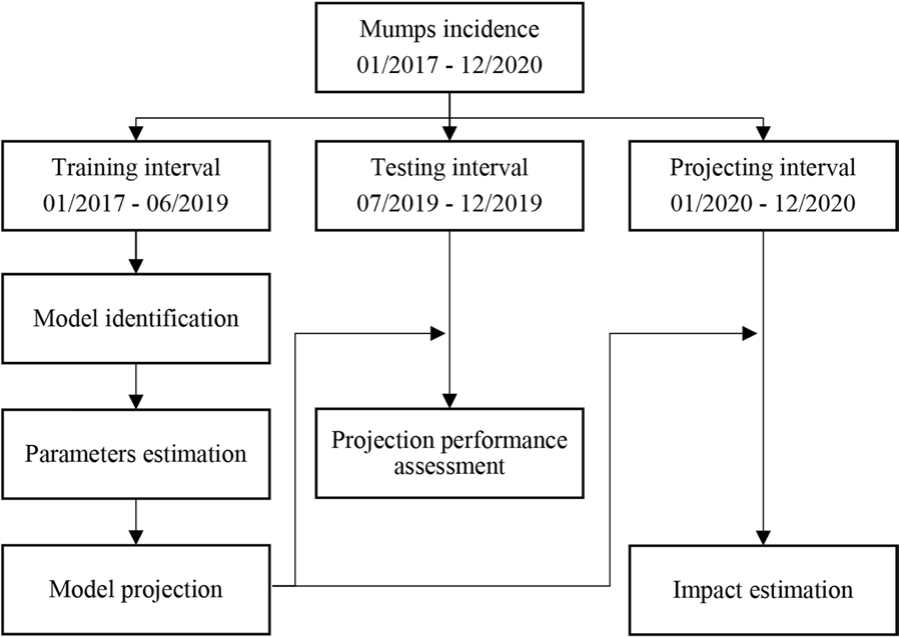
Flowchart on the modelling process

Figure 1 showed the modelling process. We divided all data into three intervals: training interval (01/2017–06/2019), testing interval (07/2019–12/2019), and projecting interval (01/2020–12/2020). The stationarity of time series was judged by the Augmented Dickey-Fuller (ADF) test, and white noise was judged by the Ljung-Box test. We estimated *d* and *D* based on the time series diagram and ADF test. Next, we identified parameters *p*, *d*, *Q*, *D* by the autocorrelation function (ACF) and partial autocorrelation function (PACF), and optimized parameters by Akaike information criterion (AIC). Finally, residuals were diagnosed as white noise by the Ljung-Box test. All analyses were done using R software version 4.0.2.

### Model prediction and assessment

We projected the monthly incidence of mumps from July 2019 to December 2020. The projecting precision of the model was assessed by calculating the mean absolute percentage error (MAPE) between the reported cases and projected cases from July 2019 to December 2019. It is calculated as follows [12]:

$$MAPE = \frac{1}{n}\sum\limits_{t = 1}^{n} {\left| {\frac{{Y_{t} - F_{t} }}{{Y_{t} }}} \right|}$$

We compared the monthly number of mumps cases reported in 2020 against the average from corresponding periods in the preceding years (2017–2019) and the projected values by the seasonal ARIMA model. We performed a monthly one-sided (less) paired difference *t*-test using R version 4.0.2[6]. We calculated the percent change with 95% confidence interval (95%*CI*) in mumps cases reported in 2020 compared with the projected values using the Wilson method [13].

## Results

### Trend of mumps incidence in Sichuan

**Fig. 2:**
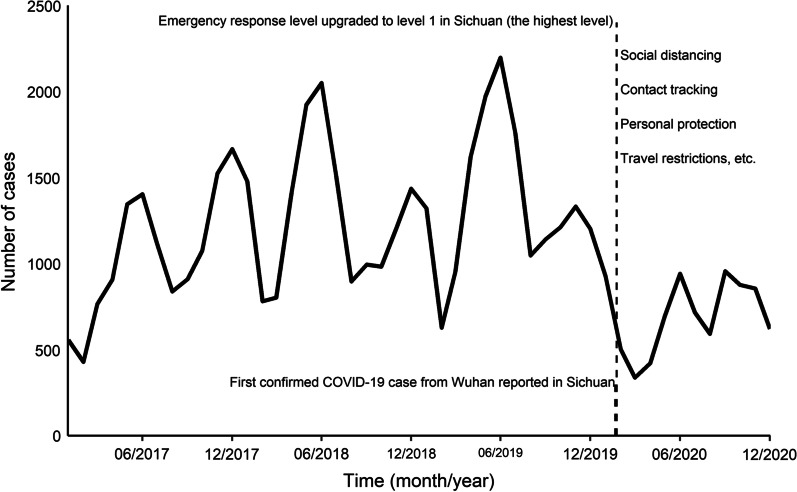
Trend of mumps incidence per month in Sichuan from 2017 to 2020

During 2017–2019, the epidemic of mumps had obvious seasonality (Fig. 2). Two seasonal peaks occurred regularly in May or June and December. Although the epidemic peaks also occurred in 2020, the monthly number of mumps cases decreased (*P* < 0.001), compared with the average from the corresponding periods in the preceding years (2017–2019).

### Model estimation and checking

The training interval is non-stationary time series (ADF *P* = 0.2148) with seasonality, which needs to be differenced (*d* = 1) and seasonally differenced (*D* = 1, *S* = 12). According to the ACFs and PACFs, we identified possible parameters, *p*, *q*, *P*, *Q*. Based on the minimum principle of AIC value (Additional file 2: Table S1), we optimized parameters and the optimal model was ARIMA (0, 1, 2) × (0, 1, 0) _12_, AIC = 236.35, given by:

$$\nabla \nabla_{12} x_{t} = (1 - 0.3983B + 0.6017B^{2} )\varepsilon_{t}$$

The diagnostics suggested there was white noise in model residues (Additional file 3: Figure S2).

### Model projection

**Fig. 3:**
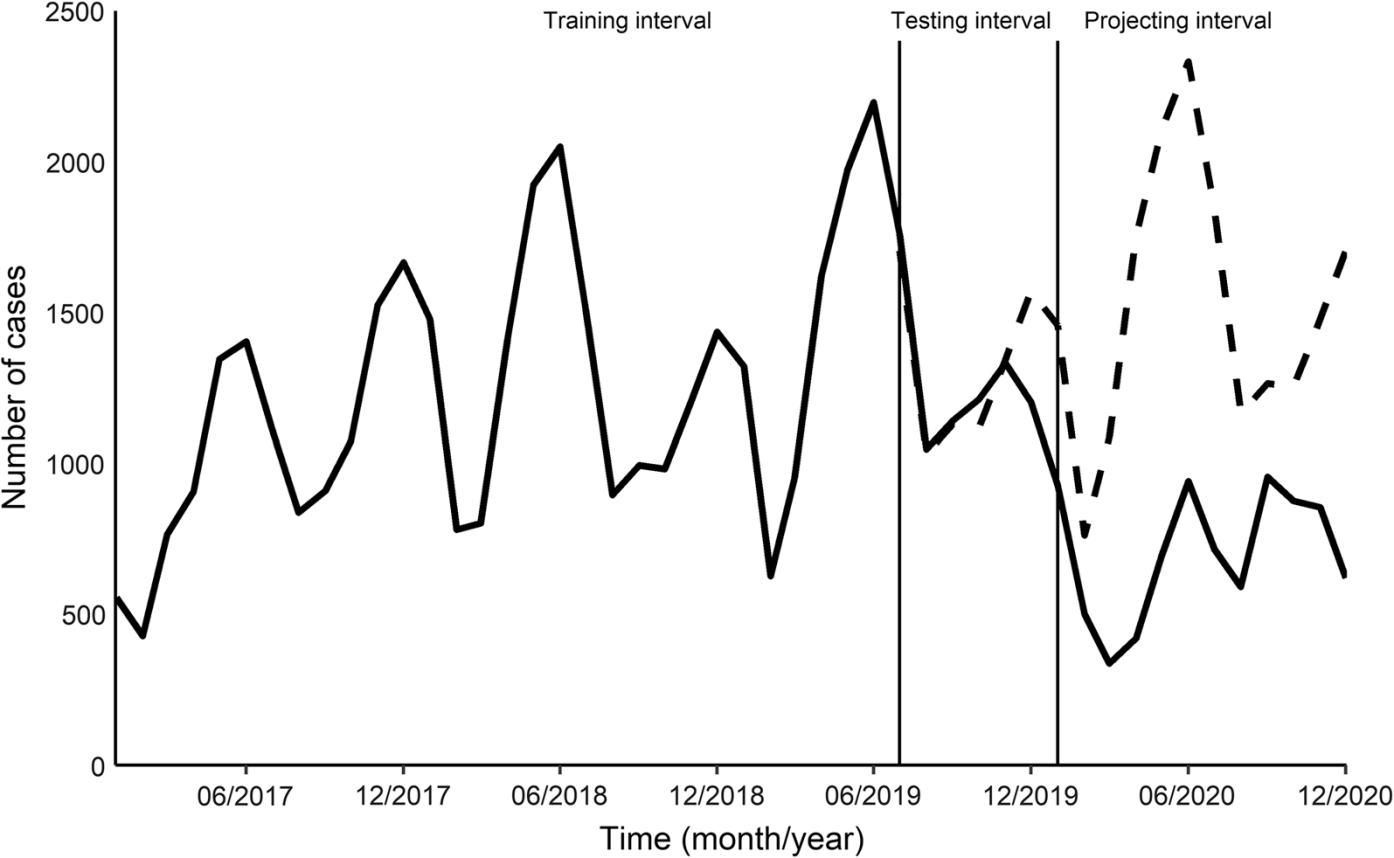
Best model projecting results (dashed line) and variation in reported mumps cases per month

We projected the mumps incidence in the testing interval. Compared with reported values, the mean absolute percentage error (MAPE) was 7.51% (Fig. 3). We also projected mumps incidence in 2020. The monthly reported number of mumps cases in 2020 decreased (*P* < 0.001), compared with the projected cases.

The percent change of the reported mumps cases in 2020 compared with projected cases varied over the course of the COVID-19 epidemic (Table 1). From January to December, the number of mumps cases was estimated to decrease by 36.3% (33.9–38.8%), 34.3% (31.1–37.8%), 68.9% (66.1–71.6%), 76.0% (73.9–77.9%), 67.0% (65.0–69.0%), 59.6% (57.6–61.6%), 61.1% (58.8–63.3%), 49.2% (46.4–52.1%), 24.4% (22.1–26.8%), 30.0% (27.5–32.6%), 42.1% (39.6–44.7%), 63.5% (61.2–65.8%), respectively. The total number of mumps cases in 2020 was estimated to decrease by 53.6% (52.9–54.3%).

**Table 1:** Comparison of projected and reported mumps cases in 2020

Month	Projected cases	Reported cases	Percent change (%) (95%*CI*)^a^
January	1456	927	− 36.3 (− 33.9 to − 38.8)
February	763	501	− 34.3 (− 31.1 to − 37.8)
March	1088	338	− 68.9 (− 66.1 to − 71.6)
April	1755	422	− 76.0 (− 73.9 to − 77.9)
May	2106	695	− 67.0 (− 65.0 to − 69.0)
June	2332	942	− 59.6 (− 57.6 to − 61.6)
July	1839	716	− 61.1 (− 58.8 to − 63.3)
August	1166	592	− 49.2 (− 46.4 to − 52.1)
September	1264	956	− 24.4 (− 22.1 to − 26.8)
October	1253	877	− 30.0 (− 27.5 to − 32.6)
November	1479	856	− 42.1 (− 39.6 to − 44.7)
December	1705	622	− 63.5 (− 61.2 to − 65.8)
Total	18,206	8444	− 53.6 (− 52.9 to − 54.3)

*CI*: confidence interval

^a^95%*CI* calculated using the Wilson method

## Discussion

This study provides evidence of a short-term effect of non-pharmaceutical interventions against COVID-19 for preventing mumps incidence in Sichuan. We estimated a 53.6% (52.9–54.3%) reduction of mumps cases in 2020.

Normalized public health measures to contain COVID-19 probably reduce the mumps transmission in 2020 because both have similarities in the modes of transmission through contact and droplet spread [9]. School closures can play an important role in mumps transmission because mumps is the most common infection in children and adolescents in China [10]. Over the long run, school closures will not continue until in autumn. After the severe acute respiratory syndrome (SARS) outbreak in 2003, there has been an uptrend in mumps incidence in China [14]. Thus, this effect can gradually attenuate unless the second-wave COVID-19 occurs.

During the COVID-19 epidemic, the effect of non-pharmaceutical interventions against COVID-19 on mumps incidence varied. Normally, school holidays start in mid-January and end in mid-February, coinciding with the COVID-19 outbreak in China. These can explain the phenomenon that the percent change of mumps cases in January and February is lower than in the next five months. Travel restrictions of regional tourism relaxed in August, schools reopened in September, person-to-person contacts increased, the effect of non-pharmaceutical interventions against COVID-19 attenuated. As the second-wave COVID-19 of Sichuan occurred and was contained in December in Pidu district, Chengdu, this effect strengthened.

Our study has several limitations. First, we projected mumps cases in 2020 using a seasonal ARIMA model based on the past data, so the prediction error is inevitable. Compared with the projected cases, the reported cases in 2020 markedly declined, coinciding with the results when compared with the average from corresponding periods in the preceding years (2017–2019). Our results still hold. Second, we could not identify the independent effect of each of the non-pharmaceutical interventions against COVID-19 on mumps incidence. Finally, we identified a marked decline in the mumps incidence, it is uncertain that such interventions can reduce the spread of other respiratory viruses. Mumps is a common childhood infection [9], school closure could effectively block close contact between children and mitigate the person-to-person transmission of mumps.

## Conclusions

We observed a substantial decline in mumps incidence in Sichuan, China, after the normalized implementation of social and public health measures against COVID-19. Our finding suggests that non-pharmaceutical interventions against COVID-19 have had a remarkable impact on mumps incidence.

## Supplementary Information

**Additional file 1: Figure S1.** A seasonal-trend decomposition diagram.

**Additional file 2: Table S1.** Seasonal ARIMA models comparison.

**Additional file 3: Figure S2.** A diagnostic plot for the final time-series fit.

## Data Availability

All data and code during the current study are available from the corresponding author on reasonable request.
