# Supplementary figures and images for: Estimating the impact of non-pharmaceutical interventions against COVID-19 on mumps incidence in Sichuan, China
Source: BMC Infect Dis. 2021 Aug 30;21:886. doi: 10.1186/s12879-021-06584-9 (PMC8404184; doi:10.1186/s12879-021-06584-9)

**
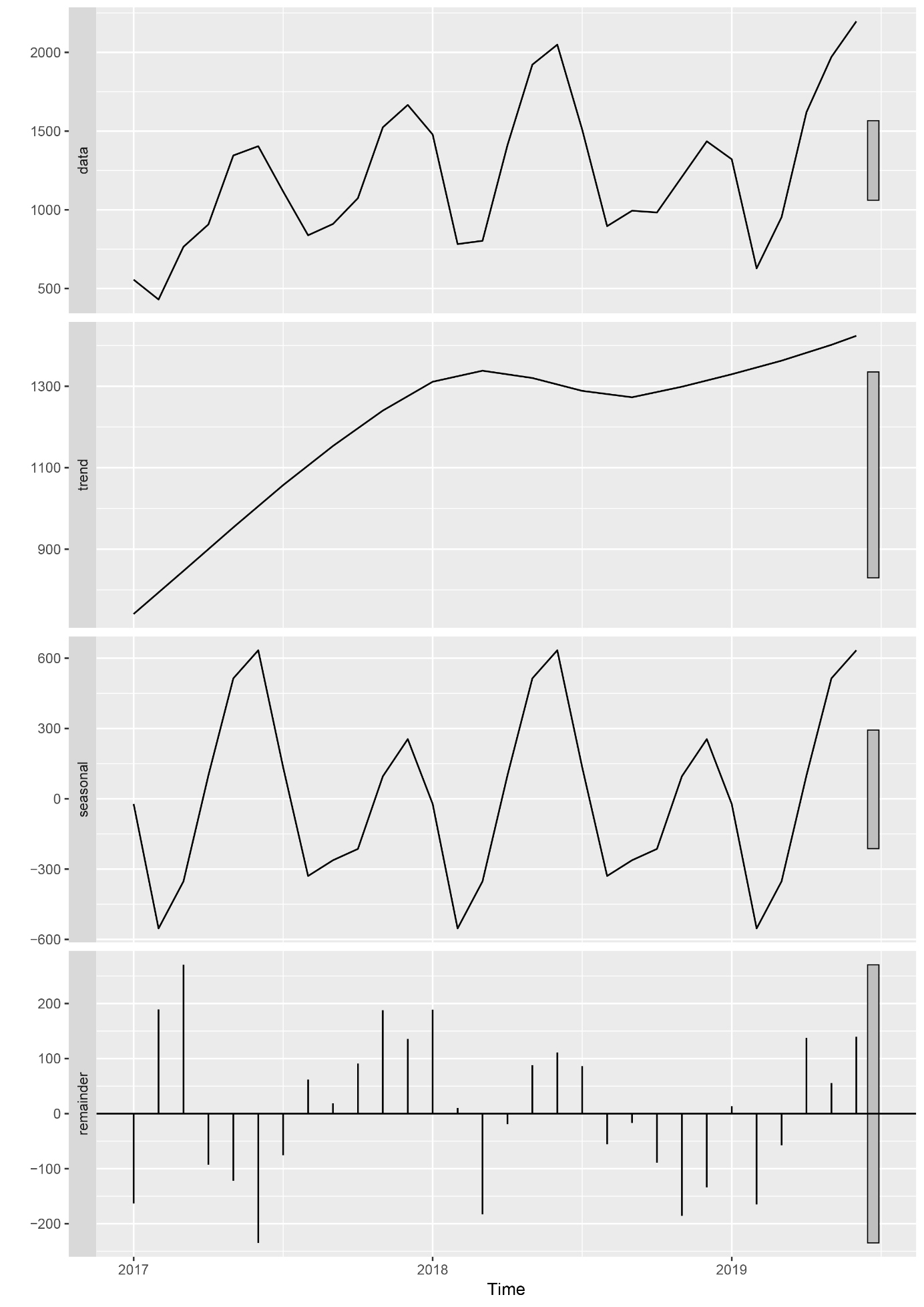
**

**Figure S1.** A seasonal-trend decomposition diagram.

Supplement: Supplementary file 1 — Additional file 1: Figure S1. A seasonal-trend decomposition diagram. [file 12879_2021_6584_MOESM1_ESM.docx]

**
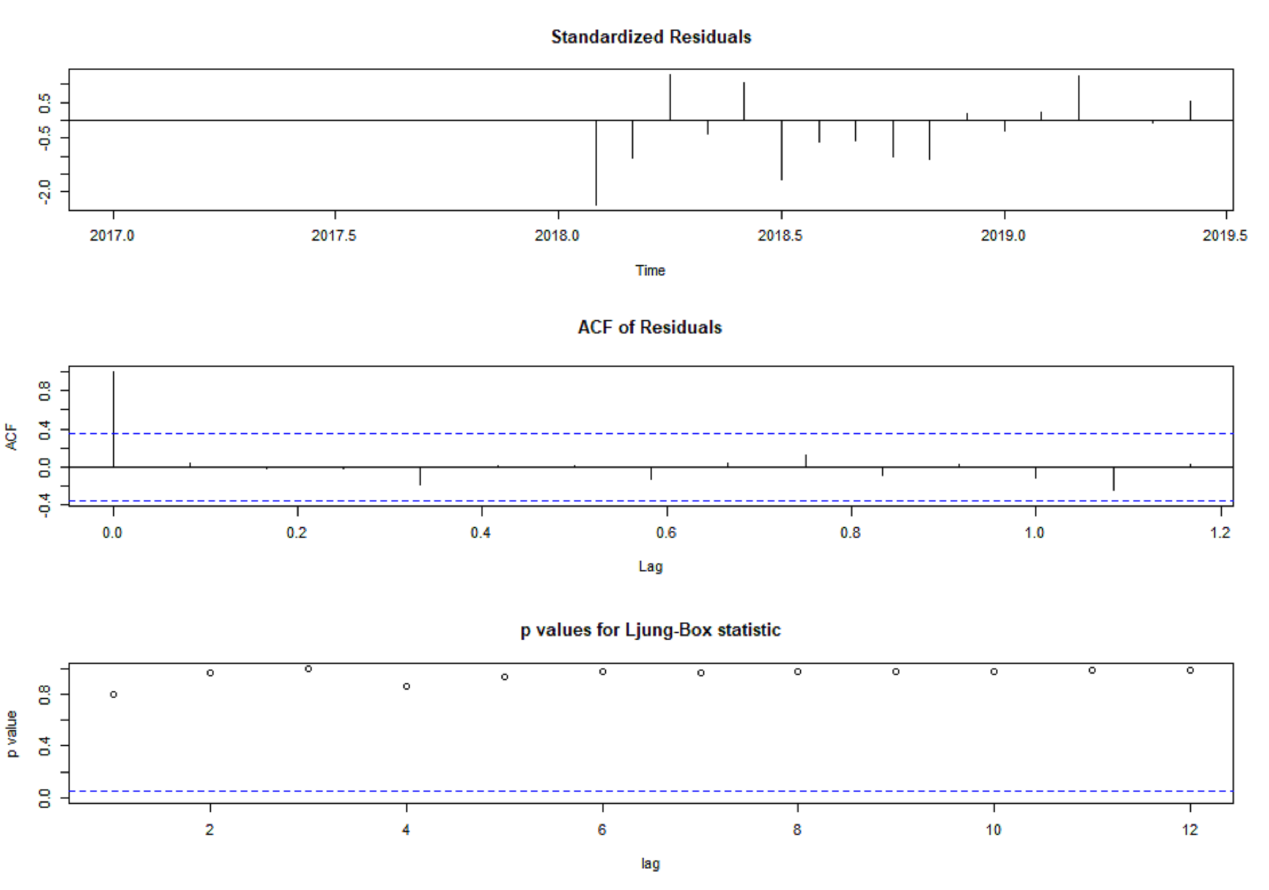
 Figure S2.** A diagnostic plot for time-series fits

Supplement: Supplementary file 3 — Additional file 3: Figure S2. A diagnostic plot for the final time-series fit. [file 12879_2021_6584_MOESM3_ESM.docx]
