# Supplementary material for: Estimating the impact of non-pharmaceutical interventions against COVID-19 on mumps incidence in Sichuan, China
Source: BMC Infect Dis. 2021 Aug 30;21:886. doi: 10.1186/s12879-021-06584-9 (PMC8404184; doi:10.1186/s12879-021-06584-9)
Supplement: Supplementary file 2 — Additional file 2: Table S1. Seasonal ARIMA models comparison. [file 12879_2021_6584_MOESM2_ESM.docx]

**Table S1.** Seasonal ARIMA models comparison.

| Model | AIC | *P* for Ljung-Box test | MAPE (%) |
| --- | --- | --- | --- |
| ARIMA (0,1,1)×(0,1,0)_12_ | 238.31 | 0.22 | 10.39 |
| **ARIMA (0,1,2)×(0,1,0)_12_** | **236.35** | **0.80** | **7.51** |
| ARIMA (1,1,0)×(0,1,0)_12_ | 239.34 | 0.98 | 8.45 |
| ARIMA (1,1,1)×(0,1,0)_12_ | 237.77 | 0.60 | 8.90 |
| ARIMA (1,1,2)×(0,1,0)_12_ | 238.34 | 0.86 | 7.53 |
| ARIMA (2,1,0)×(0,1,0)_12_ | 239.05 | 0.99 | 8.88 |
| ARIMA (2,1,1)×(0,1,0)_12_ | 238.20 | 0.49 | 7.44 |
| ARIMA (2,1,2)×(0,1,0)_12_ | 240.25 | 0.61 | 7.29 |
| ARIMA (0,1,1)×(1,1,0)_12_ | 240.29 | 0.22 | 13.08 |
| ARIMA (0,1,2)×(1,1,0)_12_ | 238.34 | 0.83 | 7.66 |
| ARIMA (1,1,0)×(1,1,0)_12_ | 241.23 | 0.94 | 12.61 |
| ARIMA (1,1,1)×(1,1,0)_12_ | 239.75 | 0.58 | 10.48 |
| ARIMA (1,1,2)×(1,1,0)_12_ | 240.34 | 0.86 | 7.49 |
| ARIMA (2,1,0)×(1,1,0)_12_ | 241.04 | 0.99 | 10.19 |
| ARIMA (2,1,1)×(1,1,0)_12_ | 240.17 | 0.49 | 8.91 |
| ARIMA (2,1,2)×(1,1,0)_12_ | 241.00 | 0.47 | 10.88 |
| ARIMA (0,1,1)×(1,1,1)_12_ | 242.29 | 0.22 | 13.08 |
| ARIMA (0,1,2)×(1,1,1)_12_ | 240.34 | 0.83 | 7.65 |
| ARIMA (1,1,0)×(1,1,1)_12_ | 243.23 | 0.94 | 12.61 |
| ARIMA (1,1,1)×(1,1,1)_12_ | 241.75 | 0.58 | 10.48 |
| ARIMA (1,1,2)×(1,1,1)_12_ | 242.34 | 0.86 | 7.49 |
| ARIMA (2,1,0)×(1,1,1)_12_ | 243.04 | 0.99 | 10.14 |
| ARIMA (2,1,1)×(1,1,1)_12_ | 242.17 | 0.49 | 8.91 |
| ARIMA (2,1,2)×(1,1,1)_12_ | 242.92 | 0.44 | 12.13 |
| ARIMA (0,1,1)×(0,1,1)_12_ | 240.29 | 0.22 | 13.08 |
| ARIMA (0,1,2)×(0,1,1)_12_ | 238.34 | 0.83 | 7.65 |
| ARIMA (1,1,0)×(0,1,1)_12_ | 241.23 | 0.94 | 12.62 |
| ARIMA (1,1,1)×(0,1,1)_12_ | 239.75 | 0.58 | 10.48 |
| ARIMA (1,1,2)×(0,1,1)_12_ | 240.34 | 0.86 | 7.49 |
| ARIMA (2,1,0)×(0,1,1)_12_ | 241.04 | 0.99 | 10.18 |
| ARIMA (2,1,1)×(0,1,1)_12_ | 240.17 | 0.49 | 8.89 |
| ARIMA (2,1,2)×(1,1,1)_12_ | 240.98 | 0.47 | 11.20 |
| ARIMA (0,1,0)×(0,1,0)_12_ | 237.79 | 0.49 | 7.75 |
| ARIMA (0,1,0)×(0,1,1)_12_ | 239.60 | 0.59 | 13.26 |
| ARIMA (0,1,0)×(1,1,0)_12_ | 239.60 | 0.59 | 13.26 |
| ARIMA (0,1,0)×(1,1,1)_12_ | 241.60 | 0.59 | 13.26 |

AIC: Akaike information criterion; MAPE: mean absolute percentage error.
